# Short- and Long-term Prognostic Impacts of Diabetes Mellitus in Patients Undergoing Off-pump Coronary Artery Bypass Grafting with Skeletonized Internal Thoracic Artery Graft

**DOI:** 10.14789/ejmj.JMJ25-0013-OA

**Published:** 2025-08-30

**Authors:** SHUNYA ONO, KAN KAJIMOTO, TAIRA YAMAMOTO, RYOMA ODA, TAKESHI KINOSHITA, ATSUSHI AMANO, MINORU TABATA

**Affiliations:** 1 Department of Cardiovascular Surgery, Juntendo University, School of Medicine, Tokyo, Japan; 1 Department of Cardiovascular Surgery, Juntendo University, School of Medicine, Tokyo, Japan

**Keywords:** off-pump coronary artery bypass grafting, diabetes mellitus, skeletonized arterial graft

## Abstract

**Objectives:**

Diabetes mellitus (DM) is a widely recognized risk factor for adverse outcomes after coronary artery bypass grafting (CABG). This study aimed to reassess the impact of DM on the short- and long-term outcomes of patients undergoing CABG using skeletonized internal thoracic artery (ITA) grafts.

**Materials:**

This retrospective analysis included 2,737 consecutive patients who underwent CABG between 2002 and 2020 at Juntendo University. The patients were divided into two groups based on the presence or absence of DM. Outcomes were analyzed for both short- and long-term periods to evaluate the prognostic impact of DM.

**Results:**

Mean follow-up was 7.1 ± 4.9 years. Diabetic patients had a higher prevalence of coexisting diseases and impaired preoperative cardiac and renal function than non-diabetic patients. While short-term outcomes, including in-hospital mortality and deep sternal wound infection, were similar between the groups, patients with diabetes exhibited higher rates of postoperative complications, such as acute kidney injury. During long-term follow-up, patients with diabetes had significantly poorer outcomes, including higher all-cause mortality, cardiac mortality, and major adverse cardiac and cerebrovascular events (MACCE).

**Conclusion:**

The use of skeletonized ITA grafts was not associated with an increased incidence of deep sternal wound infection (DSWI), even in patients with diabetes. DM remains a strong risk factor for both short- and long-term adverse outcomes in patients who undergo CABG. These findings emphasize the need for improved perioperative and long-term management of patients with diabetes to achieve better CABG outcomes.

## Introduction

Coronary artery bypass grafting (CABG) has demonstrated superior long-term outcomes compared with percutaneous coronary intervention (PCI) with drug-eluting stents in patients with diabetes, as shown by landmark trials such as the SYNTAX (Synergy between Percutaneous Coronary Intervention with TAXUS and Cardiac Surgery) and FREEDOM (Future Revascularization Evaluation in Patients with Diabetes Mellitus) trials^1, 2)^. Despite these benefits, diabetes mellitus (DM) remains a strong risk factor for adverse outcomes after CABG. Previous studies have documented the negative effects of DM on long-term prognosis following CABG^3-5)^.

Recent studies have reported that use of the bilateral internal thoracic arteries (BITA) may improve long-term outcomes in patients with diabetes. However, there have been concerns that using BITA in patients with diabetes could increase the risk of deep sternal wound infection (DSWI). Recently, harvesting arterial grafts using the skeletonization technique has been shown to reduce this risk even in patients with diabetes^6)^.

This study aimed to evaluate the short- and long-term prognostic impacts of DM in patients undergoing off-pump CABG with skeletonized internal thoracic artery (ITA) grafts as part of a modern surgical strategy that reflects contemporary techniques and real-world outcomes.

## Methods

### Ethics statement

All study protocols were approved by the Research Ethics Committee of Juntendo University Faculty of Medicine on March 6, 2025 (approval no. E24- 0493). Given the retrospective design of the study, the requirement for informed consent was waived. The study was conducted in accordance with the ethical principles outlined in the Declaration of Helsinki.

### Study design and inclusion criteria

Management of this database was performed according to institutional ethics policies and was approved by an internal review board. This was a single-center retrospective cohort analysis using prospectively collected data from a comprehensive institutional database. This study included consecutive patients who underwent off-pump CABG between 2002 and 2020. The patients were divided into two groups based on the presence or absence of DM. Patients with a history of cardiac surgery were excluded from analysis. The primary endpoints of the study were short-term outcomes, including in-hospital complications, and long-term outcomes, including all-cause mortality, cardiac mortality, and the incidence of major adverse cardiac and cerebrovascular events (MACCE).

### Patient data and follow-up

Patient data, including preoperative characteristics, operative data, and postoperative outcomes, were obtained from the Juntendo CABG database. Remote outcomes were collected by serial contact (every five years) with patients or their families until November 2020.

### Definitions

DM was defined as a fasting plasma glucose level ≥ 126 mg/dl or fulfilling at least one of the following conditions: hemoglobin A1c ≥ 6.1% or treatment with antidiabetic agents (insulin or oral hypoglycemic drugs). Hypertension was defined as systolic blood pressure ≥ 140 mmHg, diastolic blood pressure ≥ 90 mmHg, or the need for treatment with antihypertensive medications. The estimated glomerular filtration rate (eGFR) was calculated using the Cockcroft-Gault equation. DSWI is defined as an infection involving the sternum and/or mediastinal tissues requiring surgical intervention, positive bacterial cultures, or prolonged antibiotic therapy.

Postoperative death was defined as death within 30 days of surgery. Postoperative stroke was defined as new stroke diagnosed using magnetic resonance imaging or computed tomography. Postoperative acute kidney injury was defined as > 50% increase in serum creatinine levels from baseline. Cardiac death was defined as death owing to myocardial infarction, congestive heart failure, arrhythmia, or sudden death. MACCE were defined as all-cause death, nonfatal myocardial infarction, heart failure requiring hospital admission, and stroke or transient ischemic attack.

### Operative procedures

All patients underwent surgery via median sternotomy or left minithoracotomy under general anesthesia. All arterial grafts were dissected using an ultraharmonic scalpel in a fully skeletonized fashion. No bone wax was used for sternal hemostasis. The arterial grafts were covered with warm papaverine hydrochloride gauze, and spasms were released with olprinone injections. In isolated CABG, off-pump coronary artery bypass (OPCAB), which involves performing CABG on a beating heart without cardiopulmonary bypass, is generally performed. In OPCAB, a suction stabilizer is used to fix the coronary artery site for anastomosis and the coronary artery is exposed. An incision was made in the coronary artery and the proximal side was snared with an elastic string to create a bloodless operative field. Internal shunt tubes were used if excessive coronary blood flow was present or if hemorrhage or myocardial ischemia was a concern. A CO_2_ blower was used to disperse any blood locally and achieve a bloodless operative field. Coronary artery anastomosis was performed using continuous 8-0 or 7-0 propylene sutures. Since 2010s, subcutaneous drains have been routinely placed and connected to continuous negative pressure during sternal closure to reduce the risk of wound complications. (Blake^®^ silicone drain and J-VAC^®^ Suction Reservoir (Ethicon, Inc., a Johnson & Johnson company, Somerville, NJ, USA)).

### Graft selection strategy

In principle, the left ITA (LITA) is used to revascularize the left anterior descending artery (LAD) when stenosis is present. If a LITA graft was unavailable because of poor condition of the vessel, the right ITA (RITA) was used for revascularization of the LAD. The RITA was used for the LCX or diagonal branch. When the length of the in situ RITA was insufficient to reach the target vessels, it was used to create a Y-composite graft with the LITA graft. When BITAs were not considered appropriate for elderly, severely obese, compromised, or frail patients, a radial artery (RA) or SVG was used for surgical revascularization of the LCX. The right gastroepiploic artery (GEA) or SVG is routinely used in surgical revascularization of the right coronary artery (RCA). If the GEA was not available due to a hostile abdomen or poor condition or if native RCA blood flow was excessive, SVG was selected for revascularization of the RCA. Sequential grafting has been aggressively used to create bypasses in multiple dependent coronary arteries using a single graft.

### Statistical analysis

Continuous variables are presented as means with standard deviations, while categorical variables are summarized as frequencies and percentages. Comparisons between the diabetic and non-diabetic groups were conducted using Student’s *t*-test for continuous variables and the χ^2^ test or Fisher’s exact test for categorical variables, as appropriate.

Survival analyses were performed using the Kaplan-Meier method, and differences between groups were assessed using the log-rank test. A multivariate Cox proportional hazards model was used to evaluate the independent impact of DM on long-term outcomes following CABG. The covariates included in the multivariate model were: age, sex, use of BITA grafts, eGFR rate < 45 mL/min/ 1.73 m^2^ (eGFR45), hypertension, history of cerebrovascular accidents, left ventricular ejection fraction < 35% (LVEF35), and the number of distal anastomoses. Differences were considered statistically significant in all analyses. Statistical calculations were performed using SPSS Statistics software (v.16.0; IBM, Armonk, NY, USA).

## Results

### Baseline characteristics

Of the 2,737 patients analyzed in this study, 1,537 (56%) had DM, and 1,200 (44%) did not. Patients with DM exhibited a higher prevalence of comorbidities such as dyslipidemia and hypertension and significantly worse preoperative renal function than non-DM patients. Comparison of baseline patient characteristics between DM and non-DM patients were shown in Table 1. DM patients also had a significantly lower left ventricular ejection fraction (LVEF) compared to non-DM patients (56 ± 14% vs. 59 ± 12%, p < 0.001), and a greater number of coronary artery lesions (3.2 ± 0.8 vs. 3.0 ± 0.9, p < 0.001). Other baseline characteristics, including age and sex distribution, were comparable between the two groups.

**Table 1:** Comparison of baseline patient characteristics between DM and non-DM patients

Variable	non-DM (N 1200)	DM (N 1537)	P-value
Age (years)	68 ± 11	67 ± 9	0.366
Male, n (%)	1041 (83.4)	1284 (81.0)	0.091
HbA1c (%)	5.4 ± 0.5	6.9 ± 1.1	< 0.001
FBS (mg/dL)	104 ± 23	145 ± 58	< 0.001
Dyslipidemia, n (%)	913 (73.2)	1214 (76.5)	0.039
Total cholesterol (mg/dL)	175 ± 39	168 ± 41	< 0.001
Triglyceride (mg/dL)	130 ± 77	134 ± 94	0.208
LDL-cholesterol (mg/dL)	102 ± 33	96 ± 33	< 0.001
HDL-cholesterol (mg/dL)	47 ± 14	44 ± 13	< 0.001
Hypertension, n (%)	899 (72.2)	1199 (75.6)	0.038
Smoking history, n (%)	736 (59.3)	1008 (63.7)	0.017
Family history, n (%)	307 (24.8)	347 (21.9)	0.079
Peripheral artery disease, n (%)	172 (13.7)	257 (16.2)	0.081
eGFR (ml/min/1.73 m^2^)	66 ± 31	61 ± 44	0.001
BNP (pg/mL)	159 ± 356	274 ± 675	< 0.001
LVEF (%)	59 ± 12	56 ± 14	< 0.001
Diseased vessels (n)	3.03 ± 0.90	3.14 ± 0.76	< 0.001
History of CVA, n (%)	165 (13.2)	291 (18.3)	< 0.001
EuroScore	2.3 ± 3.1	2.8 ± 3.7	0.002
JapanScore	2.3 ± 4.6	2.7 ± 5.1	0.036

Abbreviations not defined in the text: HbA1c, hemoglobin A1c; FBS, fasting blood sugar; LDL, low-density lipoprotein: HDL, high-density lipoprotein; BNP, brain natriuretic peptide; CVA, cerebrovascular accident; LVEF, left ventricular ejection fraction.

### Operative parameters and short-term results

Operative parameters and short-term outcomes are presented in Table 2. Operative strategies differed between DM and non-DM patients, with DM patients more frequently receiving arterial grafts, such as BITA and GEA grafts. The average number of distal anastomoses was higher in DM patients (3.6 ± 1.3 vs. 3.3 ± 1.3, p < 0.001). Despite these differences in operative parameters, in-hospital mortality was low and comparable between DM and non-DM patients (1.1% vs. 0.9%, p = 0.70). The observed in- hospital mortality in both groups was lower than the predicted mortality rates estimated by the EuroSCORE and JapanSCORE. There was no significant difference in the incidence of DSWI between DM and non-DM patients (2.3% vs. 1.6%, p = 0.17). However, patients with DM had a higher incidence of postoperative complications, including acute kidney injury (7.1% vs. 4.1%; p = 0.001). The rates of stroke, respiratory failure, and DSWI were similar between the groups.

**Table 2:** Comparison of operative parameters and postoperative outcomes between DM and non-DM patients

Variable	non-DM (N 1200)	DM (N 1537)	P-value
Operative parameters			
Urgent/emergent, n (%)	104 (8.3)	108 (6.8)	0.126
SITA	1209 (96.9)	1533 (96.7)	0.747
BITA	683 (54.7)	960 (60.5)	0.002
GEA	849 (68.0)	985 (62.1)	0.001
SVG	536 (42.9)	751 (47.4)	0.019
Number of distal anastomoses (n)	3.3 ± 1.1	3.5 ± 1.2	0.009
Postoperative outcomes			
Inhospital mortality, n (%)	14 (1.1)	20 (1.2)	0.862
Low output syndrome, n (%)	14 (1.1)	32 (2.0)	0.061
PMI, n (%)	15 (1.2)	15 (0.9)	0.509
POAF, n (%)	350 (29)	360(23)	0.001
Stroke, n (%)	10 (0.8)	23 (1.5)	0.192
Respiratory failure, n (%)	131 (10.5)	136 (8.6)	0.086
Acute kidney injury, n (%)	51 (4.1)	112 (7.1)	0.001
Deep sternal wound infection, n, %	5 (0.4)	11 (0.70)	0.327
Surgical site infection, n (%)	8 (0.6)	22 (1.3)	0.064
ICU stay (days)	2.0 ± 6.4	2.1 ± 6.9	0.748
Hospital stay (days)	11 ± 13	12 ± 11	0.108

Abbreviations not defined in the text: BITA, bilateral internal thoracic artery; GEA, gastroepiploic artery; SVG, saphenous vein graft; ICU, intensive care unit; PMI, perioperative myocardial infarction; POAF, postoperative atrial fibrillation.

### Long-term results

**Figure 1:**
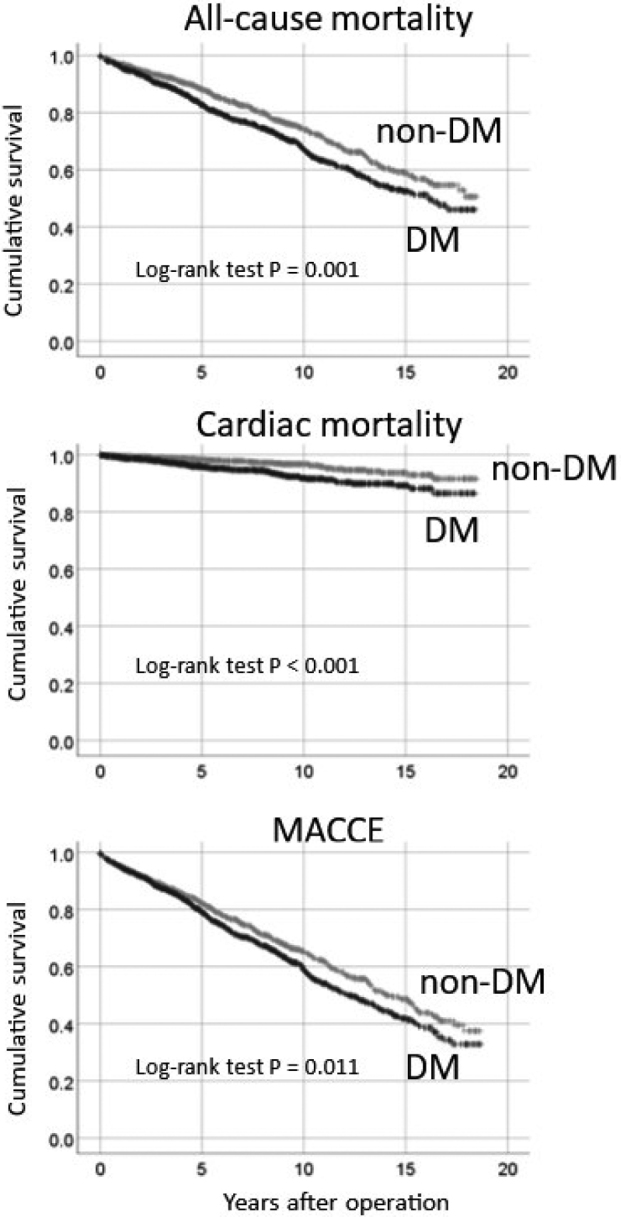
Kaplan-Meier cumulative survival curves comparing DM and non-DM patients were analyzed using the logrank test

The mean follow-up period was 7.1 ± 4.9 years, with a follow-up rate of 96%. During the follow-up period, Kaplan-Meier survival analysis showed that patients with DM had significantly higher rates of all-cause mortality, cardiac mortality, and MACCE than patients without DM (Figure 1). Multivariate Cox proportional hazards analysis further indicated that DM was associated with poorer outcomes (Table 3). DM patients had a higher risk of all- cause mortality (HR: 1.30, 95% CI: 1.11-1.52, p < 0.001) and cardiac mortality (HR: 1.86, 95% CI: 1.24-2.80, p < 0.001). Additionally, DM patients had a significantly increased risk of MACCE (HR: 1.18, 95% CI: 1.03-1.35, p = 0.020).

**Table 3:** Results of multivariate Cox proportional hazards regression analysis

	All-cause death			Cardiac death			MACCE		
	HR	95%CI	*P*	HR	95%CI	*P*	HR	95%CI	*P*
DM	1.30	1.11-1.52	< 0.001	1.86	1.24-2.8	< 0.001	1.18	1.03-1.35	0.02
Age (years)	1.06	1.05-1.07	< 0.001	1.02	1-1.04	0.14	1.04	1.03-1.04	< 0.001
Gender (male)	0.61	0.49-0.75	< 0.001	0.89	0.56-1.42	0.63	0.72	0.6-0.86	< 0.001
BITA	0.78	0.66-0.92	< 0.001	1.16	0.78-1.73	0.46	0.90	0.79-1.04	0.16
eGFR<45	2.62	2.22-3.08	< 0.001	4.63	3.16-6.78	< 0.001	2.31	1.99-2.68	< 0.001
HT	0.96	0.8-1.15	0.63	1.01	0.65-1.58	0.96	0.95	0.81-1.11	0.53
History of CVA	1.39	1.15-1.66	< 0.001	1.61	1.06-2.46	0.03	1.33	1.12-1.57	< 0.001
LVEF>35%	0.81	0.51-1.29	0.37	0.48	0.17-1.32	0.15	0.77	0.52-1.16	0.21
Number of distal anastomosis	0.99	0.93-1.05	0.79	1.04	0.9-1.2	0.63	0.93	0.89-0.99	0.01

Abbreviations not defined in the text: BITA, bilateral internal thoracic artery; eGFR, estimated glomerular filtration rate; HT, hypertension; CVA, cerebrovascular accident; LVEF, left ventricular ejection fraction.

## Discussion

This study revealed that DM remains a significant predictor of adverse outcomes in patients undergoing CABG, despite advancements in surgical technology. CABG is widely recognized as the most effective revascularization strategy for multivessel coronary artery disease (CAD) has demonstrated excellent short-term outcomes in both diabetic and non-diabetic patients. However, despite improvements in perioperative management and surgical technology and techniques, patients with diabetes have significantly worse long-term outcomes, including higher rates of all-cause and cardiac mortality as well as MACCE. In addition, proportional hazard analysis revealed that a reduced eGFR and lower LVEF were independent predictors of long-term adverse outcomes. These findings suggest that the increased prevalence of renal dysfunction and impaired cardiac function in patients with diabetes may contribute to a poorer prognosis. However, the predictive value of reduced LVEF and eGFR as independent risk factors suggests that these conditions contribute significantly to adverse outcomes, regardless of the presence of DM, and should therefore be carefully considered in all patients undergoing CABG. This study represents one of the largest analyses of CABG outcomes in diabetic patients, with a mean follow-up period of 7.1 years and a sample size of 2,737 patients. These findings highlight current strategies for treating DM and the necessity for development of both surgical technology and postoperative treatment.

DM is not only a key risk factor for the progression of CAD, but also an independent predictor of adverse outcomes following revascularization. Flather et al. demonstrated a 20-30% increase in long-term mortality among diabetic patients undergoing CABG compared with non-diabetic patients^7)^. Similarly, Bundhun et al. reported that patients with diabetes exhibited higher rates of cardiac mortality and MACCE^8)^. This study reaffirms the critical impact of DM, as patients with diabetes demonstrated a higher prevalence of baseline comorbidities such as dyslipidemia, hypertension, and impaired renal function. Additionally, diabetic patients had a significantly lower LVEF and a greater number of CADs than their non-diabetic counterparts. These risk factors, combined with the systemic effects of DM, likely contribute to the observed differences in long-term outcomes.

CABG remains the ‘gold standard’ for revascularization in patients with multivessel CAD, particularly those with DM, as supported by landmark trials such as SYNTAX and FREEDOM. However, this study demonstrated that even CABG, the most comprehensive revascularization approach, cannot fully eliminate the risks associated with DM. In our cohort, patients with DM accounted for 56% of the population, reflecting a significant burden of DM in contemporary CABG practice. The findings also indicate the effectiveness of modern surgical techniques, including off-pump CABG, and the frequent use of arterial grafts such as BITA and GEA grafts. These strategies, which are known to enhance long- term graft patency and clinical outcomes, were widely used in this study. Suzuki et al. reported that the use of multiple arterial grafts reduces the adverse effects of DM in terms of patient outcomes^9)^. Similarly, Alsaleh et al. reported that arterial grafting improved the long-term survival of patients with diabetes who underwent CABG^10)^. Several studies have compared the outcomes of off- and conventional on-pump CABG. The Veterans Affairs Randomized On/Off Bypass Study (ROOBY) trial demonstrated that off-pump CABG is associated with worse long-term outcomes, including lower graft patency and higher mortality rates, than on-pump CABG^11)^. In contrast, the CORONARY trial reported that off-pump CABG achieved long-term outcomes comparable to those of on-pump CABG, suggesting that careful patient selection can mitigate some of the disadvantages associated with off-pump techniques^12)^. In this study, in-hospital mortality was low and comparable between diabetic (1.2%) and non-diabetic patients (1.1%), significantly lower than the predicted mortality rates based on the EuroSCORE and JapanSCORE. These findings indicate the excellent quality of the surgical treatment provided to the patients in this cohort. However, diabetic patients are more prone to several complications, such as acute kidney injury, emphasizing the need for targeted perioperative management in DM patients.

Another concern in patients with DM after CABG is the increased risk of DSWI. However, in this study, the incidence of DSWI was not significantly different between DM and non-DM patients. One possible explanation for this favorable outcome is the standardized infection prevention strategy implemented at our institution. Specifically, bone wax was not used for sternal hemostasis, which may have helped reduce the risk of infection. Additionally, all ITA grafts were harvested in a fully skeletonized fashion, a technique previously shown to minimize the risk of DSWIs in patients with diabetes^6)^. Moreover, at the time of sternal closure, a subcutaneous suction drain was routinely emplaced and connected to a negative-pressure reservoir system to reduce fluid accumulation and promote wound healing. The combination of these strategies may have contributed to the low and comparable incidence of DSWI between the groups, even in the high-risk diabetic population.

The findings of this study highlight the limitations of surgical treatment alone in addressing the long-term risks associated with DM. Although innovations such as multiple arterial grafting and off-pump techniques have enhanced outcomes, comprehensive postoperative management is equally critical. Optimal medical treatment (OMT) is essential to mitigate the persistent adverse effects of DM. These include optimal control of hyperglycemia, dyslipidemia, and hypertension, along with lifestyle modifications, such as smoking cessation, weight management, and dietary improvements.

Recent evidence suggests that integrating newer glucose-lowering agents, such as sodium-glucose cotransporter-2 inhibitors (SGLT2i) and glucagon-like peptide-1 receptor agonists (GLP-1 RA), into postoperative care protocols may offer additional cardiovascular benefits^13)^. Enhanced perioperative strategies, including stricter glucose monitoring, prehabilitation programs, and management of complications such as acute kidney injury, could further improve long-term outcomes for diabetic patients undergoing CABG.

## Limitations

This study was limited by its retrospective, single- center design and absence of randomization. Additionally, angiographic data regarding graft patency were not evaluated, and the applicability of these results to a broader population requires further external validation. Although multivariate regression was used to adjust for confounding variables, the possibility of residual confounding factors could not be fully excluded. Moreover, data concerning perioperative and long-term glycemic control were not available, precluding analysis of the influence of glycemic management on clinical outcomes. In addition, subgroup analyses based on the severity of DM (e.g., insulin dependence or glycemic burden) were not conducted, which may have provided additional prognostic insights. Finally, we lacked follow-up data on glycemic control and the achievement of OMT, both of which are known to affect long-term outcomes after CABG. This limits our ability to assess their impact on prognosis.

## Conclusion

This study highlights the ongoing challenges in determining optimal treatment strategies for patients with DM undergoing CABG. Although modern surgical techniques have markedly improved short- term outcomes, DM remains a major risk factor for adverse long-term events, including mortality and MACCE. Importantly, in this study, the incidence of DSWI did not increase in patients with DM, which may be attributed to the use of skeletonized BITA harvesting and other standardized infection control protocols at our institution. These findings underscore the potential benefits of combining advanced surgical techniques with individualized perioperative management to reduce complications in high-risk populations. Continued advancement of surgical approaches and comprehensive secondary prevention remain essential to further improve outcomes in patients with DM undergoing CABG.

## Author contributions

SO analyzed and interpreted patient data and was a major contributor to writing the manuscript. KK contributed to the study design and critically revised the manuscript. YT was a major contributor to the acquisition of data for this study. RO contributed to ethical approval. All authors have read and approved the final manuscript.

## Conflicts of interest statement

The authors declare that there are no conflicts of interest.

## Supplementary Material

**Supplemental Table 1:** Components of MACCE in DM and non-DM Groups

	non-DM (N 1200)	DM (N 1537)
All-cause death, n (%)	278 (23)	401 (26)
Non fetal myocardial infarction	8 (0.7)	7 (0.5)
Heart failure requiring hospital admission	43 (3.6)	68 (4.4)
Stroke or transient ischemic attack	65 (5.4)	59 (3.8)
